# Effects of *Withania somnifera* on Cortisol Levels in Stressed Human Subjects: A Systematic Review

**DOI:** 10.3390/nu15245015

**Published:** 2023-12-05

**Authors:** Matteo Della Porta, Jeanette A. Maier, Roberta Cazzola

**Affiliations:** Department of Biomedical and Clinical Sciences, University of Milano, 20157 Milan, Italy; jeanette.maier@unimi.it (J.A.M.); roberta.cazzola@unimi.it (R.C.)

**Keywords:** ashwagandha, stress, adaptogens, adrenal function, withanolides

## Abstract

Background: Withania somnifera (WS), a popular medicinal plant of the Solanaceae family, contains active ingredients with antioxidant, anti-inflammatory, immunomodulatory, and anti-stress activities. However, its precise mechanisms of action and optimal use as a supplement are not yet fully understood. The objective of this systematic review is to assess the impact of WS supplementation on cortisol levels in stressed humans by analyzing clinical trials conducted prior to May 2023. Methods: The assessment was carried out following the guidelines of Preferred Reporting Items for Systematic Reviews and Meta-Analyses (PRISMA) by exploring the databases of EMBASE, PubMed, Google Scholar, CENTRAL, and Scopus. Results: Of the 4788 articles identified, only 9 studies met the selection criteria. The selected studies varied in terms of design, results, formulations, dosages, and treatment duration (30–112 days), and involved subjects with varying degrees of stress. WS supplementation decreases cortisol secretion with no significant adverse effects. Nonetheless, none of the studies evaluated the potential impact of cortisol reduction on adrenal function and long-term effects. Conclusions: Brief-term supplementation with WS appears to have a stress-reducing effect in stressed individuals. However, since the long-term effects of WS supplementation are not yet fully understood, WS supplements should be used under medical supervision.

## 1. Introduction

In recent decades, the use of herbal medicinal products has gained enormous popularity around the world. These products are sold on the Internet and in health stores, but many are not rigorously tested for their effectiveness and safety through clinical trials. *Withania somnifera* (L.) Dunal (WS), also known as ‘Ashwagandha’ in Sanskrit and as ‘Indian ginseng’ in Ayurveda, is a very popular medicinal plant of the Solanaceae family. It has been used in traditional medicine for more than 2500 years [1]. WS is described in the Indian Ayurvedic medicine system and referred to as an important herb in the traditional Unani and Chinese medicinal systems. This ayurvedic herb has been used to treat a variety of diseases such as arthritis, anxiety, insomnia, tuberculosis, asthma, and fibromyalgia [2]. However, the logic of Ayurvedic treatments is based on complex algorithms for combining herbs. These treatments aim at restoring the balance of the physiological functions in the body and are administered in accordance with the constitution of the individual as well as the dynamic response to the treatments. On the contrary, WS supplements on the market are generally composed of a fraction of the active ingredients of this herb. Currently, WS supplements are promoted especially for stress and anxiety, sleep, male infertility, and athletic performance. With the increasing public use of WS, there is a growing need to understand its biological properties to validate and optimize its use. WS has been the subject of extensive research into its pharmacological activities, but only a select few have been validated. In animal models, WS has shown anti-microbial, anti-tumor, anti-stress, neuroprotective, cardioprotective, and anti-diabetic activities (revised in [3]). Moreover, the administration of WS seems to enhance male fertility treatment [4] and aid the regulation of thyroid hormone levels in individuals with subclinical hypothyroidism [5], and it might offer neurological benefits, such as cognitive promotion [6]. Meanwhile, in vitro studies have shown the molecular basis for its antioxidant and anti-inflammatory activity [7,8,9]. However, all these bioactivities need validation in vivo and their mechanism of action requires additional studies.

WS is also known for its adaptogenic activity [3,8], i.e., the ability to increase the state of nonspecific resistance to stress and to decrease sensitivity to stressors, resulting in protection from stress and the prolongation of the resistance phase [10,11]. There are many systems in the body that individually or collectively regulate the level of stress, including the hypothalamic–pituitary–adrenal axis (HPA axis), the autonomic nervous system (ANS), and the immune system. The HPA axis reacts to stressors by secreting cortisol from the adrenal cortex into the circulation. Adaptogens also exert their activity through a reduction in adrenal activity [10]. However, prolonged stress and sustained HPA activation cause cortisol resistance and the initiation of several pro-inflammatory pathways, including the activation of the nuclear factor (NF)-κB pathway [12] leading to several chronic health conditions [13]. On the other hand, an inappropriate reduction in adrenal activity could lead to hypoadrenalism with serious health consequences due to the inability to respond to acute stress, such as illness or severe infection. 

WS supplements typically contain root, leaf, or root/leaf extracts. The active ingredients are concentrated mainly in the roots of the plant and, to a lesser extent, in the leaves and stems; however, their concentration in WS products varies depending on whether they are extracted [10,14]. In addition, the composition of these substances is influenced by various factors, such as the growth phase of the plant, and the geographic location and season during which the samples are collected [15]. The major active ingredients of WS are withanolides, a group of steroids naturally occurring in the Solanaceae family, and alkaloids that exhibit a wide range of biological activities [16]. WS’s pharmacological activities are attributed mainly to its two main withanolides, withaferin A and withanolide D [17,18,19,20]. Most of the WS-based supplements on the market are titrated in withanolides. Among the various alkaloids, withanine is the main constituent. In addition, different classes of sitoindosides, saponins, phenolic compounds, flavonoids, triethylene glycol, and many other secondary bioactive metabolites of the plants with a broad spectrum of therapeutic activity were isolated and characterized [10,14]. Therefore, WS is a reservoir of bioactive compounds. A recent review by Tetali et al. lists about 140 of these compounds [21] and highlights the presence of a complex group of withanolides, which occur also as glycosides (withanosides). Preclinical studies suggest that withanolides act as anti-inflammatory agents by inhibiting lymphocyte proliferation and the complement system [22]. In addition, some of these compounds display anti-cancer activity through the inhibition of cyclooxygenase-2 and the suppression of proliferation in various tumor cell lines, as well as the hindrance of angiogenesis [23]. Certain withanolides possess reactive thiol groups that could exhibit antioxidant effects and/or bind with other electrophile sensors, thereby regulating transcriptional or post-transcriptional responses [15].

The potential mechanisms by which WS bioactive compounds may affect cortisol secretion have not yet been fully clarified. Withaferin A might affect circulating cortisol levels presumably owing to a direct interaction with glucocorticoid receptors (GR) in the brain [23], but other WS bioactive substances could also modulate the levels of this hormone [24]. The effect on cortisol levels of WS may be also an indirect effect of its sedative and hypnotic activities. The HPA axis response to stress can also be regulated by gamma-aminobutyric acid (GABA) signaling. A high-quality sleep, with the correct duration of the slow wave sleep and the rapid eye movement (REM) phase, led a reduction in the release of cortisol during the daytime [25]. In human studies, WS has a clear activity in the regulation of sleep and in the increase in the quality of life of people with nonrestorative sleep, insomnia, and anxiety [26,27,28] via GABA modulation [29]. In addition, the methanolic extract of the WS root demonstrated a GABA mimetic activity when tested on GABA-A rat brain channels micro-transplanted into Xenopus oocytes [30]. Furthermore, it was observed that the aqueous root extract of WS had a hypnotic effect on mice. This effect was believed to be caused by the interaction between GABA receptors and certain water-soluble components. Notably, this effect is observed only with the aqueous root extract and not with withaferin A and withanolide A [31]. It is believed that the induction of sleep by WS involves water-soluble components that regulate GABAergic activity. The extract obtained from the leaves of WS, which are rich in triethylene glycol, has been shown to enhance non-REM sleep and mildly impact REM sleep in mice. Triethylene glycol, when administered alone, has similar effects that depend on the dosage. [14,24].

Extensive research has been conducted to examine and analyze the diverse biological impacts of WS on stress in humans [32]. Plasma cortisol levels are frequently used as a stress biomarker in studies involving humans. This paper aims to provide a detailed and comprehensive systematic review that focuses on the effectiveness of WS in reducing cortisol levels in stressed human subjects.

## 2. Materials and Methods

### 2.1. Search Strategy

Literature searches were conducted using the following databases: Pubmed, EMBASE, CENTRAL, Google Scholar, and Scopus. The search query was the following: ((withania somnifera) OR (winter cherry)) OR (Indian ginseng)) OR (ashwagandha)) AND ((cortisol) OR (stress)) OR (hypocortisolaemic effect)). The search was performed on 16 May 2023 using the PRISMA Guidelines 2020 [33]. The systematic review was registered on PROSPERO database with the ID: CRD42023475797. The search was limited to peer-reviewed original articles published in English language.

### 2.2. Study Selection

#### 2.2.1. Inclusion Criteria

Papers were included based on the following criteria:

1. Original peer-reviewed articles
2. Human studies (randomized controlled trials, prospective or retrospective cohort study, or cross-sectional)
3. Studies on adult population (≥18 years)
4. Studies including healthy population.
5. Studies with the only use of WS.
6. Studies including the measurement of cortisol level.

#### 2.2.2. Exclusion Criteria

The articles with the following characteristics were excluded:

1. Not human studies (cell-based and animal or plant studies).
2. Review, case report, and congress abstract.
3. Studies including multi-herbal and/or multi-mineral preparations.
4. Studies include pharmacological treatments.
5. Studies without measurements of cortisol level.

### 2.3. Data Extraction

Title and abstract screening of all articles initially found in the search strategies was performed independently by two reviewers (RC, MDP). Data were extracted manually by a single reviewer. 

## 3. Results

### 3.1. Study Selection

**Figure 1:**
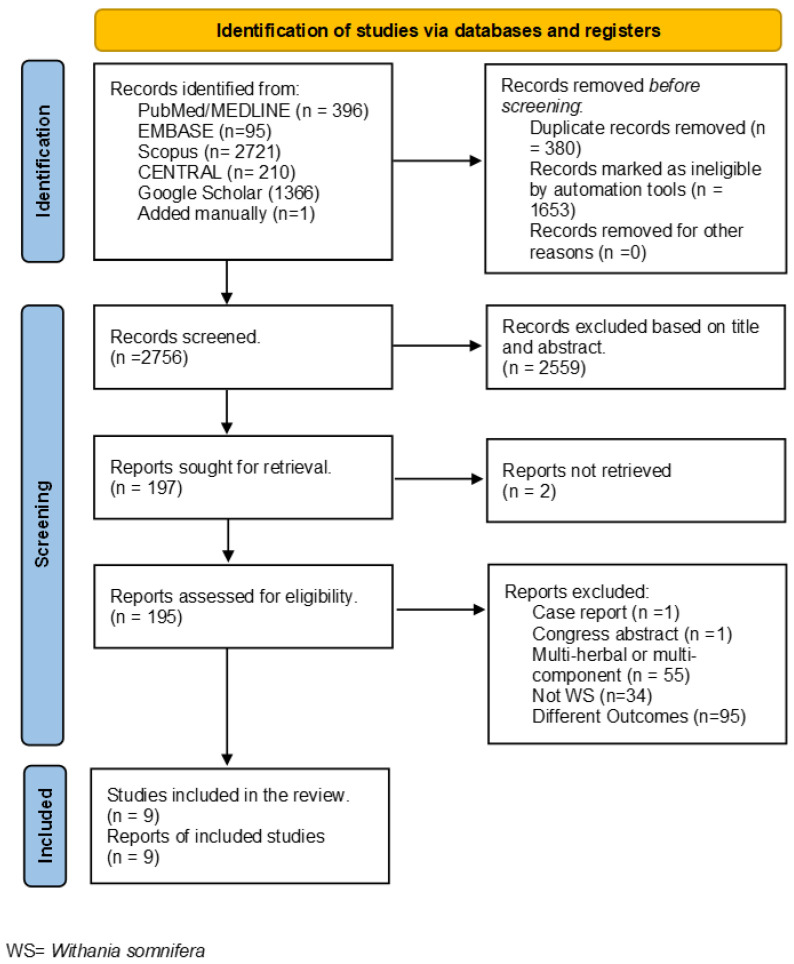
PRISMA Flow Diagram of study selection, WS = *Withania somnifera*.

The literature search identified 4788 articles from PubMed/MEDLINE, EMBASE, CENTRAL, Google Scholar, and Scopus, respectively, and 396, 95, 210, 1366, 2721, and 1 papers were detected by a manual search of the authors. A total of 2033 records were removed before the screening, of which 380 papers were duplicate articles; the other 1653 were removed by automation tools (search filters for removing the follow type of documents “Review”; “Systematic review and/or meta-analysis”; “Book chapter”; “Conference paper”; “Editorial”; “Short Survey”; “Note”; ”Letter”; ”Erratum”; “Retracted”; and “Studies not involving human subjects)). In total, 2756 records were screened, and 2559 records were excluded based on title and abstract reading. Of the 197 reports identified, 2 were not considered because it was not possible to retrieve the full text. The full-text reading led to the exclusion of 186 articles because they were case reports (*n* = 1), Congress abstract (*n* = 1), multi-herbal or multi-component (*n* = 55), the medicinal plant analyzed was not WS (*n* = 34), and had a different outcome (*n* = 95). Nine articles were included in this systematic review. Figure 1 reported the PRISMA flow diagram.

### 3.2. Results of Individual Studies

The nine articles included in this systematic review are summarized in Table 1. Eight studies are randomized double blind studies [34,35,36,37,38,39,40,41] and one is a controlled study [42]. Eight had a parallel design [34,35,36,37,38,39,40,42] and one is a cross-over [41]. The nine studies included healthy subjects with mental stress assessed through various questionnaires, e.g., Profile of Mood State (POMS), Perceived Stress Scale (PSS), Hamilton Rating Scale for Anxiety (commonly termed HAM-A), Depression, Anxiety and Stress Scale (DASS), and the World Health Organization-Five Well-Being Index (WHO-5). The effect of WS on cortisol secretion was determined by measuring hormone levels in plasma in seven studies [34,35,36,37,38,40,42] and saliva in two studies [39,41]. In the study of Chandrasekhar et al. [34] (study 1), a WS full spectrum extract, standardized at 5% withanolides, was tested in an RCT involving 61 healthy subjects with high levels of stress measured with a WHO-5 questionnaire. The extract was administered twice a day at a dose of 300 mg for 60 days. The extract reduced the plasmatic cortisol level by 27.9% from baseline. This extract was tested in two additional RCT double blind parallel studies performed by Choudhary et al. (study 2) [35] and Salve et al. (study 3) [36]. In study 2, an identical posology was used. The treatment involved 50 healthy subjects with high levels of stress measured with PSS > 20 and lasted 56 days. The WS extract reduced plasmatic cortisol by 22.7% from baseline together with a slight reduction in body weight [35]. In study 3, the extract was given to 58 healthy subjects with PSS > 20, but with a different posology, 125 mg twice a day in the first group and 300 mg twice a day in the second group. The result shows a dose-response effect because in the first group the decrease in plasmatic cortisol was 16.5%, and in the second group, it was 32.6%. In addition, the subjects taking the higher dose of WS root extract showed a significant reduction in anxiety as assayed through the HAM-A [36]. In study 4 [42], Mahdi et al. assayed 5 g of WS root extract without standardization in a controlled trial without placebo in normo-spermatic (NS) subjects. The authors tested the WS root extract in three groups of NS subjects: infertile, smokers, and stressed in parallel with one group of control NS subjects. The duration of the treatment was 90 days, after which plasma cortisol was decreased in the three groups (infertile, smokers, and stressed), with respect to the control group, by 11%, 28%, and 32%, respectively. The other four studies used extracts from WS root and leaves [37,38,39,41]. In study 5 and study 6 [37,41], an ethanol/water 70:30 extract of WS leaves and roots (35% withanolide glycosides) was administered. In study 5 [37], WS extract was tested in 30 stressed healthy subjects versus 30 placebo-controlled subjects, at the daily dose of 240 mg for 60 days. At the end of the treatment the plasma cortisol level decreased by 23.39% from baseline, and an improvement in HAM-A and DASS was observed. Moreover, testosterone levels significantly increased by 11% and dehydroepiandrosterone (DHEA) levels significantly decreased by 8% [37]. In study 6 [41], 60 mg of the same WS extract were administered for 56 days to healthy overweight subjects with POMS above 50th percentiles in a RCT cross-over trial. The results indicated no changes in salivary cortisol, while salivary testosterone and DHEA levels increased, respectively, by 14.7% and 18%. In study 7 [38], an extract of WS leaves and roots in water, standardized in 11.90% withanolide glycosides, 1.05% withaferin A, and 40.25% oligosaccharides, was administered in increasing doses, up to 250 mg two times a day for 60 days. The WS extract reduced plasma cortisol up 30.5% from baseline in a dose-dependent manner. Moreover, the increase in serum DHEA and the decrease in C reactive protein, fasting blood glucose, and total cholesterol were reported [38]. In study 8 [39], an extract of WS leaves and root (3.5% withanolides) was administered at 225 mg or 400 mg a day for 30 days, and, surprisingly, only the lower doses decreased salivary cortisol. Finally, in study 9 [40], a WS butanol/water extract (5% withanolides) was administered at the daily dose of 300 mg for 90 days. The treatment decreased serum cortisol by 29.87% from baseline, without significant effects on Brain-Derived Neurotrophic Factor (BDNF) [40]. 

Since cortisol secretion shows a regular circadian rhythm in healthy subjects, we also analyzed the time of hormone measurements in the different studies. As shown in Table 2, the cortisol levels were measured mainly in the morning. In studies 1 [34], 3 [36], 5 [37], and 8 [39], the cortisol levels were measured in the morning, but the time was not specified. In study 2 [35], the time of cortisol measurement was not specified. On the contrary, in studies 4 [42], 6 [41], 7 [38], and 9 [40], cortisol was measured at 8.00 a.m., 6.00–8.00 a.m., 9.00–11.00 a.m., and 9.00–11.00 a.m., respectively. In all studies, the time zone is not indicated. In addition, the time of WS administration varied from study to study: after dinner [37,41], before lunch [38], after breakfast [40], or after food ingestion [34,36], while it was not indicated in others [35,39,42].

The data of safety assessments are depicted in Table 3. The adverse events (AEs) of WS supplementation were registered in only six studies. Out of these, four studies [36,37,40,41] reported no side effects of WS, while only two studies [34,35] reported mild AEs. Subjects with adverse events made up six out of thirty (20%) in study 1, and 4% in study 2. In the first study, the main AEs were rhinitis (3.3%), constipation (3.3%), cough and cold (3.3%), decreased appetite (3.3%), and sleepiness (3.3%) [34]. In the second study, no significant differences in vital parameters, such as systolic blood pressure, diastolic blood pressure, pulse rate, respiratory rate, and body temperature, were observed between the placebo group and the treated group. The main mild AEs reported by 2 subjects out of 52 (4%) were giddiness, heavy head, blurred vision, and gastric hyperacidity [35]. The safety analysis was performed at the end of the treatment with a Patient’s Global Assessment of Tolerability to Therapy (PGATT) test questionnaire [35] or by a reported AEs during the visit with the physicians [34]. In study 3, the authors stated that clinical safety and tolerability of the interventions were measured by analyzing the significant changes in the vital parameters and the biochemical parameters assayed; however, the only biochemical parameter measured was serum cortisol concentrations [36]. In study 5, safety was assessed by the evaluation of complete blood count (red blood cell, white blood cell, hemoglobin, hematocrit, platelets, and erythrocyte sedimentation rate) and a lipid profile (low-density lipoprotein, cholesterol, high-density lipoprotein, triglycerides, and very-low-density lipoprotein) [37]. No significant AEs were reported by participants [37]. Differently, in study 6, the authors do not claim to have evaluated safety and did not measure any blood chemistry parameters They reported that WS was well tolerated with no significant differences in AEs between the placebo and active drug treatment groups [41]. However, it should be emphasized that this treatment did not influence cortisol salivary levels (Table 1). Finally, in study 9, the safety was assessed as the proportion of subjects who discontinued the study treatment due to AEs, such as changes in vital parameters and laboratory investigations (complete blood count, alanine aminotransferase, aspartate aminotransferase, and serum creatinine levels).

In agreement with previous human studies (reviewed in [43]), these findings seem to indicate that there were no major negative effects or significant changes in important health indicators such as blood counts, organ function, or vital signs in the short term (30–112 days). Taken together, the results suggest that WS can lower the plasma cortisol level in stressed healthy individuals by 11% to 32.63%, without any significant self-reported AEs in six out of nine studies. However, it should be noted that the lack of proper consideration of circadian rhythms of cortisol secretion may have affected these results, as well as that safety, when considered, was evaluated by routinary blood chemistry analyses without considering HPA function.

## 4. Discussion

This systematic review explores the adaptogenic effect of WS, focusing on the secretion of the glucocorticoid hormone cortisol. Study design and results varied between the nine selected studies. The overall result of these studies was that WS decreased cortisol secretion. Amongst the reviewed studies, the supplementation of WS decreased the level of cortisol in all the seven studies in which its plasma levels were measured. This decrease varied from 11% [42] to 32.63% [36]. In contrast, the two studies in which cortisol levels were measured in saliva led to contradictory results (Table 1). However, Lopresti et al. [41] found no effect on salivary cortisol levels by administering WS after dinner, rather than in the morning. 

Stress is a common experience that every human faces. Our bodies’ response and ability to adapt to stressors are crucial for maintaining good health. This process begins with the activation of the sympathetic branch of the autonomic nervous system, followed by the HPA axis [44]. The activation of the splanchnic nerve results in the release of catecholamines, such as epinephrine and norepinephrine, which bind to α and ß-adrenergic receptors. This bond triggers catabolic pathways and leads to an increase in respiration, blood pressure, and heart rate. [44]. Furthermore, the activation of the splanchnic nerve indirectly triggers the secretion of cortical hormones from the marrow. The HPA axis processes stressors and leads to the production of adrenocorticotropic hormone (ACTH) from the pituitary gland by inducing the expression of the corticotropin-releasing hormone (CRH) in the hypothalamus. ACTH is released in the bloodstream and binds to its receptor, stimulating the production of glucocorticoids and mineralocorticoids in the adrenal cortex. Cortisol downregulates the blood concentration of CRH and ACTH via different negative feedback mechanisms [44]. The secretion of cortisol and other glucocorticoids from the adrenal gland is dynamically regulated throughout the day [45]. These hormones are released in higher amounts at the beginning of the active phase of the circadian cycle. This daily increase in glucocorticoids is caused by changes in the release of ACTH and adrenal sensitivity to ACTH. These changes are driven by the circadian timekeeping system, which is a collection of clocks located throughout the brain and body [46]. The circadian rhythm of cortisol is closely associated with the sleep–wake cycle [47] and meal-induced cortisol stimulation [48]. Cortisol was only measured at a specific time (8:00 a.m.) in Madi’s study [42], while in the other studies, it was either determined during a specific time window (9:00–11:00 in the morning) or was not specified (see Table 2). Therefore, in eight of the nine selected studies, the observed changes in cortisol levels could be due not only to WS supplementation, but also to fluctuations related to the circadian rhythm of this hormone. In addition, the timing of the administration of WS varied significantly across the nine studies considered. WS was administered after food ingestion in some studies [34,36,37,40,41], while in others, it was given away from meals [38]. It should be noted that taking supplements along with food could potentially reduce the bioavailability of the phytochemical compounds present in WS.

The exact mechanisms by which WS affects cortisol secretion are currently not fully understood. Glucocorticoids work by binding to the glucocorticoid receptor (GR), which is a member of the nuclear receptor superfamily. The GR is usually found in the cytoplasm with chaperone proteins, such as heat shock proteins [49]. When cortisol binds to the GR, it causes the chaperone proteins to dislocate, allowing the GR–cortisol complex to move to the nucleus. Once there, it binds to DNA and regulates gene expression, thus controlling various biological processes [50]. The GR can regulate gene expression even when not bound to DNA. This mechanism is known as transrepression and involves the inhibition of gene transcription induced by other transcription factors, including NF-κB, a major transcription factor for proinflammatory cytokines. The GR–cortisol complex interferes with this transcription factor through protein–protein interactions. Finally, glucocorticoid action is also mediated by membrane receptors such as the G-protein coupled receptor and its intracellular second messengers [51]. Withanolides, like other phytosterols, may act as GR receptor ligands. It has been shown that withaferin A can stably bind to GR, promoting its translocation into the nucleus in different types of cultured cells [23]. This results in an anti-inflammatory effect via a transrepression mechanism. During short-term stress, the heightened sensitivity of GR helps to manage inflammation more effectively. However, in the case of chronic stress, cortisol levels can remain elevated over an extended period, thus resulting in steroid resistance, which is marked by a decrease in the number of GRs or a reduced capability of these receptors to bind their ligands. As a result, inflammation increases triggered by NF-κB activation, leading to disrupted GR expression and function, GC resistance, and uncontrolled inflammation [52,53]. This, in turn, intensifies the stress-reactive system [54]. Hence, the withanolide-rich extracts tested in the considered studies may have indirectly reduced cortisol levels by reducing inflammation.

WS treatment was administered to subjects with varying degrees of stress in ways that differed in formulations, dosages, and treatment duration. Adverse events were considered in only six studies (Table 3). However, out of the reviewed studies, it was found that only two of them provided information on the type of mild adverse events that occurred, and the methods used to measure them. Studies on the tolerability and safety of WS are scarce and refer to short periods of administration, but data on the effects of long-term supplementation are lacking. In addition, as in the reviewed studies, these data are obtained without considering HPA function. WS is generally considered to be safe when taken in the short term (up to 3 months) [43]. The most reported AEs were mild and temporary, such as sleepiness, stomach discomfort, and diarrhea. However, other, less common, side effects have been reported, including dizziness, hallucinations, coughing, decreased appetite, nausea, and weight gain [reviewed in ref [43]. In addition, hepatotoxicity [55,56] and thyrotoxicosis in hypothyroidism [57] related to WS supplementation have been reported in humans. Moreover, a decrease in cortisol levels can be interpreted both as a positive and an adverse effect of WS supplementation, depending on its consequences on adrenal function. Recently, Fry et al. reported a case of adrenal hypofunction associated with ten weeks of WS supplementation [58], which was reversed to normal function after the discontinuation of this herbal product. The patient’s thyroid function, as well as basic hematological and biochemical parameters, were normal in this study. However, to assess the adrenal response, the Synacthen test was performed. This test measures serum cortisol levels before and after the injection of synthetic adrenocorticotropin hormone (also known as tetracosactrin). The patient showed a subnormal adrenal response to tetracosactrin. This reduction in adrenal function could potentially result in serious health consequences for the patient, such as an inability to respond to acute stress like severe illness or infection. Currently, there is no other literature available on the dynamic endocrine testing of adrenal function during WS supplementation. This highlights the need for more comprehensive reporting of adverse events in research studies. 

Since there is no other published literature on adrenal function among people taking WS, it is impossible to determine whether this product affects adrenal function differently depending on age, gender, health status, and body composition (lean body mass versus fat body mass). 

## 5. Conclusions

WS has a thousand-year history of use in traditional Indian medicine. Currently, its readily available supplement use is continuously growing. Therefore, it becomes necessary to scientifically evaluate and document both the efficacy and safety of this plant in humans. WS contains a variety of active ingredients that possess numerous properties. Supplementation with WS for a period ranging from 30 to 112 days appears to have a stress-reducing effect by lowering cortisol levels in stressed individuals. However, it is important to note that none of the studies considered in this review have evaluated the potential impact of this reduction in cortisol on adrenal function; future studies are necessary to elucidate this issue. Since WS can potentially impact the levels of other hormones and the long-term effects of its supplementation are not yet completely understood, it is advisable to consume WS supplements only under the guidance of a medical professional.

## Figures and Tables

**Table 1:** Summary of the studies.

Study	Number of Subjects *	Type of Study	Conditions	Type of Extract	Dose/ Posology	Treatment Duration (Days)	Effects	Comments
Study 1 Chandrasekhar et al. 2012 [34]	61 (WS = 30; P = 31)	RCT DB parallel	Healthy subjects Higher level of stress (score WHO-5 < 15)	WS root extract full spectrum (5% withanolides)	300 mg/b.i.d	60	↓ Plasma cortisol level by 27.9% from baseline	
Study 2 Choudhary et al. 2017 [35]	50 (WS = 25; P = 25)	RCT DB parallel	Healthy subjects with PSS ≥ 20	WS root extract full spectrum (5% withanolides)	300 mg/b.i.d	56	↓ Plasma cortisol level by 22.7% from baseline	
Study 3 Salve et al. 2019 [36]	58 (WS1 = 19; WS2 = 20; P = 20)	RCT DB parallel	Healthy subjects with PSS ≥ 20	WS root extract full spectrum (5% withanolides)	125 mg/b.i.d. WS1; 300 mg/b.i.d. WS2	56	↓ Plasma cortisol level by 16.5% and 32.63% from baseline, respectively, in WS1 and WS2	Reduction in HAM-A only for WS2 treatment
Study 4 Mahdi et al. 2011 [42]	121 (WS = 60; C = 60)	CT without P, parallel	Healthy men with 24 ≥ mHAM-A ≤ 42. NS men, NS infertile men; NS-stressed men; NS-smoker men	WS root powder	5 g/die	90	↓ Plasma cortisol level in infertile NS, NS-smokers, NS-stressed, respectively, by 11%, 28%, and 32%	
Study 5 Lopresti et al. 2019 a [37]	60 (WS = 30; P = 30)	RCT DB parallel	Healthy subjects with 6 ≥ HAM-A ≤ 17	WS leaves and roots ethanol/water 70:30 extract (35% withanolide glycosides)	240 mg/die	60	↓ Plasma cortisol level by 23.39% from baseline	↑ testosterone and DHEA level, respectively, by 11% by 8% from baseline
Study 6 Lopresti et al. 2019 b [41]	43 (WS = 23; P = 20)	RCT DB Cross-over	Healthy overweight subjects POMS above 50th percentiles	WS leaves and roots ethanol/water 70:30 extract (35% withanolide glycosides)	60 mg/die	112 (56 + 56)	No significant changes in salivary cortisol	↑ salivary testosterone and DHEA level, respectively, by 14.7% and 18%
Study 7 Auddy 2008 et al. [38]	98 (WS1 = 19; WS2 = 30; WS3 = 34; P = 15)	RCT DB parallel	Healthy subjects with 24 ≥ mHAM-A ≤ 42	WS leaves and roots water extract (11.90% withanolide glycosides; 1.05% withaferin A; 40.25% oligosaccharides)	125 mg/q. d. WS1; 125 mg/b.i.d. WS2; 250 mg/b.i.d. WS3	60	↓ Plasma cortisol level by 14.5%, 24.2 and 30.5 from baseline, respectively, in WS1, WS2 and WS3	↑ serum DHEA and decrease in CRP; FBG; and TC
Study 8 Remenapp 2021 et al. [39]	57 (WS1 = 19; WS2 = 19; P = 19)	RCT DB parallel	Healthy subjects with PSS ≥ 14	WS leaves and root extract (3.5% withanolides)	225 mg/die WS1; 400 mg/die WS2	30	↓ salivary cortisol only for WS1 group	Improvement of cognitive ability
Study 9 Gopukumar 2021 et al. [40]	125 (WS = 62; P = 63)	RCT DB parallel	Healthy subjects with 14 ≥ PSS ≥ 24	WS Butanol/water extract (5% withanolides)	300 mg/die	90	↓ Plasma cortisol level by 29.87% from baseline	No change in serum BDNF

b.i.d = bis in die; BDNF = Brain-Derived Neurotrophic Factor; C = control group; C = controls; DASS = Depression, Anxiety, and Stress Scale; DB = Double-Blind; DHEA = Dehydroepiandrosterone; FBG = Fasting Blood Glucose; HAM-A = Hamilton Anxiety Rating Scale; hs-CRP = high sensitive C-Reactive Protein; IL-6 = Interleukin-6; NS = normozoospermatic; P = placebo; PANSS = Positive and Negative Syndrome Scale; POMS = Profile of Mood State; PSS = Perceived Stress Scale; RCT = Randomized Controlled Trial; TC = Total Cholesterol; WS = Withania somnifera; WS1,2,3 = Withania Somnifera extract at different dosage, for dosage see Dose/Posology column in Table 1; * = per-protocol population.

**Table 2:** Time of cortisol levels measurement and WS administration.

Study	Time of Cortisol Measurement	Time of WS Administration
1 [34]	Morning, not specified	2 times a day, after food, not specified
2 [35]	Not specified	No controlled
3 [36]	Morning, not specified	2 times a day, after food, not specified
4 [42]	8.00 a.m.	Not specified
5 [37]	Morning, not specified	After dinner, not specified.
6 [41]	6.00–8.00 a.m.	After dinner, not specified.
7 [38]	9.00–11.00 a.m.	Before lunch, not specified
8 [39]	Morning not specified.	No controlled
9 [40]	9.00–11.00 a.m.	After breakfast, not specified

**Table 3:** Safety assessment of WS supplementation.

Study	Safety Assessment	Adverse Events (AEs)	Subjects with AEs (%)
1 [34]	Yes	Mild	20%
2 [35]	Yes	Mild	4%
3 [36]	Yes	None	0%
4 [42]	No	----	----
5 [37]	Yes	None	0%
6 [41]	Yes	None	0%
7 [38]	No	----	----
8 [39]	No	----	----
9 [40]	Yes	None	0%

## Data Availability

Data sharing is not applicable to this article.
